# Continuous chest compressions: encouraging but unusual

**DOI:** 10.1186/1757-7241-18-20

**Published:** 2010-04-16

**Authors:** Daniel Bergum, Eirik Skogvoll

**Affiliations:** 1 Department of Anaesthesiology and Emergency Medicine, St. Olav University Hospital, Trondheim, Norway; 2 Norwegian University of Science and Technology, Faculty of Medicine, Trondheim, Norway; 3 Norwegian Air Ambulance Foundation (SNLA), Drøbak, Norway

## 

In the case report by Steen-Hansen [1], the author presents evidence that continuous chest compressions may provide adequate circulation over a long time. Together with the compression-caused airway flow, circulation was sufficient to provide a critical amount of oxygen delivery to vital organs until arrival of emergency rescuers to perform eventual defibrillation. Gasping and moaning is sometimes observed while doing CPR, suggesting that it is effective in providing cerebral blood flow. Here, this notion is strengthened by the observation that small pauses of chest compressions led gasping to cease.

This case report is thus an encouraging reminder that simple, effective compression-only CPR can sometimes yield excellent results, illustrating what has been named "cardiac only resuscitation" [2]. The case report further demonstrates an essential issue in emergency medical documentation (and research) with particular relevance to cardiac arrest, namely the usefulness of time continuous registration of clinical signs and treatment. The entire episode was covered, from phone call, during CPR with the detailed description of bystander CPR until arrival of rescuers.

It is another issue, however, to what extent one may generalize to the entire population of victims of cardiac arrest.

As the author notices, it might be a feature of this particular patient and situation. Some cases of CA seem to be therapy resistant while others with apparently similar aetiology respond promptly. Patients are known to be heterogeneous, with a number of more or less favourable prognostic factors. It is further reasonable to speculate that anatomical and physiological differences (chest wall rigidity, diastolic filling between compressions, and maintenance of an open airway) will influence the effectiveness of the chest compressions.

Finally, this case could only have been a primary cardiac arrhythmia. Collapse was preceded by chest pain, the patient noted the irregularity himself, it was witnessed, and VF was confirmed at rescuer arrival. One could thereby essentially exclude non-cardiac causes that necessitate proper ventilations to restore circulation. Under all circumstances an unusual situation.

In every case of cardiac arrest, one should consider the aetiology and potential reversible factors. This may very well matter: in a recent observational study on paediatric cardiac arrest, conventional CPR was associated with better outcome [3]. It will be a most challenging task for the Emergency Medical Communication Centre to sort out these issues before providing telephone instructions [4].
